# Screening of phosphate-solubilizing bacteria and their abilities of phosphorus solubilization and wheat growth promotion

**DOI:** 10.1186/s12866-022-02715-7

**Published:** 2022-12-09

**Authors:** Zhonghua Wang, Huihong Zhang, Lu Liu, Shaojian Li, Jiufeng Xie, Xia Xue, Ying Jiang

**Affiliations:** 1 College of Resources and Environment, Henan Agricultural University, No. 63 Nongye Road, Zhengzhou, 450002 People’s Republic of China; 2 College of Environmental Science and Engineering, Beijing Forestry University, Beijing, 100089 People’s Republic of China; 3 Institute of Plant Protection, Henan Academy of Agricultural Sciences, Zhengzhou, 450002 People’s Republic of China; 4 College of Life Sciences, Henan Agricultural University, Zhengzhou, 450002 People’s Republic of China; 5 Henan Key Laboratory of Helicobacter Pylori and Microbiota and Gastrointestinal Cancer, Marshall Medical Research Center, The Fifth Affiliated Hospital of Zhengzhou University, Zhengzhou, 450002 People’s Republic of China

**Keywords:** Phosphate fertilizer, Indole-3-acetic acid, Phosphate-solubilizing bacteria, Soil phosphorus fractionation, Wheat growth

## Abstract

**Background:**

Phosphate-solubilizing bacteria (PSB) can enhance plant growth and phosphorus (P) solubilization, it also has been reported to reduce the negative effects of overused agricultural fertilizer in farmland and protect the soil environment. However, the mechanism behind this interaction has not been fully elucidated.

**Results:**

In this study, we screened out *Pseudomonas moraviensis*, *Bacillus safensis*, and *Falsibacillus pallidus* which can both solubilize P efficiently and produce indole-3-acetic acid (IAA) from sandy fluvo-aquic soils. The yield of wheat (*Triticum aestivum*) under PSB inoculation significantly increased up to 14.42% (*P* < 0.05) compared with the control treatment in phosphate fertilizer-used farmland. Besides promoting wheat growth, we found the labile P fraction in soil was significantly increased by over 122.04% (*P* < 0.05) under PSB inoculation compared with it in soils without, in parallel, the stable P fraction was significantly reduced by over 46.89% (*P* < 0.05). Furthermore, PSB inoculation increased the soil microbial biomass and activity, indicating that PSB screened out in this work performed a remarkable ability to colonize the soils in the wheat field.

**Conclusion:**

PSB from sandy fluvo-aquic soil improve wheat growth and crop productivity by increasing the labile P fraction and IAA content in the greenhouse and wheat field. Our work provides an environment and economy-friendly bacterial resource that potentially promotes sustainable agricultural development in the long term.

**Supplementary Information:**

The online version contains supplementary material available at 10.1186/s12866-022-02715-7.

## Introduction

Phosphorus (P) is one of the essential elements in plant growth and nutrient cycling in soil systems [1]. Although the quantity of the total P in soils is high, most P is not available for plants [2]. Hedley et al. [3] raised a method (modified by Tiessen and Moir [4]) that identified organic and inorganic P into three fractions including labile P (LP), moderately labile P (MLP), and stable P (SP). LP fraction refers to a fast-cycling P pool that is available for the plant in the short-term, MLP fraction refers to a slow-cycling pool can be converted into LP fraction under the specific chemical condition, SP fraction is the almost unavailable for the plant [5, 6].

To obtain sufficient P for grain production, P fertilizer is applied to most farmlands, even though its efficiency of P uptake by plants appears low as 10–25% mostly due to P fixation or loss from soils [7–9]. Phosphate anions bind to metal cations in soils easily which results in an extremely low content of soil available P (AP) for the demands of plants [9, 10]. Moreover, most of the unused P from fertilizer would be transferred into groundwater in various forms [8, 11], while P left in the soil enters the water body with the runoff, causing P fertilizer pollution [11]. The P fertilizer pollution can spread out of farmland to a broad range of natural ecosystems and returns to even more poor nutrient resources in soils [12]. Due to the low utilization of P fertilizer application, the P loss poses a lack of phosphate on plant growth and nutrient cycling in multiple ecosystems [13]. P fertilizer pollution has drawn strong attention all over the world. To date, multiple strategies have been raised to overcome or reduce its damage to soil ecosystems [14, 15], such as reducing P fertilizer application, optimization of planting system, appropriate intercropping, and developing environment-friendly fertilizers [16].

Phosphate-solubilizing bacteria (PSB) play an essential role in P cycling and promoting plant growth by increasing its P uptake in rhizosphere soils. Most PSB produces indole-3-acetic acid (IAA) which enables plant cells to grow, RNA/protein synthesis thus increasing plant growth [17]. Moreover, the microbial metabolites and low molecular weight organic acids released along with the metabolic processes of PSB are essential for P solubilization in soils [18, 19]. The effective application of PSB can release the accumulated P left by traditional P fertilizer in soils and avoid the environmental damages that result in soil hardening and water eutrophication [20, 21]. It has been proved that inoculation with PSB in farmland can increase the efficiency of P fertilizer by incorporating it with mineral and organic P [21–24]. Most studies of PSB in solubilizing P and promoting the usage of P fertilizer have been conducted in the pot experiments, it is challenging to evaluate the ability of P solubilization in the field under complex environmental conditions.

Our study aims to reveal the role of PSB in solubilizing P, enhancing P fertilizer efficiency, and promoting plant growth. In this study, we isolated PSB that can both solubilize P and produce IAA from sandy fluvo-aquic soil. We evaluated the effects of PSB on plant growth, and soil P cycling in pot experiments. Furthermore, we tested the ability of PSB on enhancing P fertilizer efficiency in fields. The present work provides a full-picture description of studying the P solubilization ability of PSB and fills the gap in understanding the mechanisms of interaction between microbes and plants in natural soil systems.

## Results

### Screening of PSB

Based on the Ca_3_ (PO_4_)_2_ solubilization and IAA production (Fig. S1), three strains (X2, X3, and X21) that showed superior ability to solubilize insoluble phosphate and produce IAA were screened out for further studies. The results showed that the three strains solubilized Ca_3_(PO_4_)_2_ in large quantities, the AP concentration in the supernatant of strains X2, X3, and X21 were 401.25, 476.60, 421.15 mg L^−1^, respectively. The IAA concentration with strains X21, X2, and X3 were 45.65, 40.42, and 34.78 μg mL^−1^, respectively.

### Morphological, physiological, biochemical, and molecular characterization of PSB

**Fig. 1:**
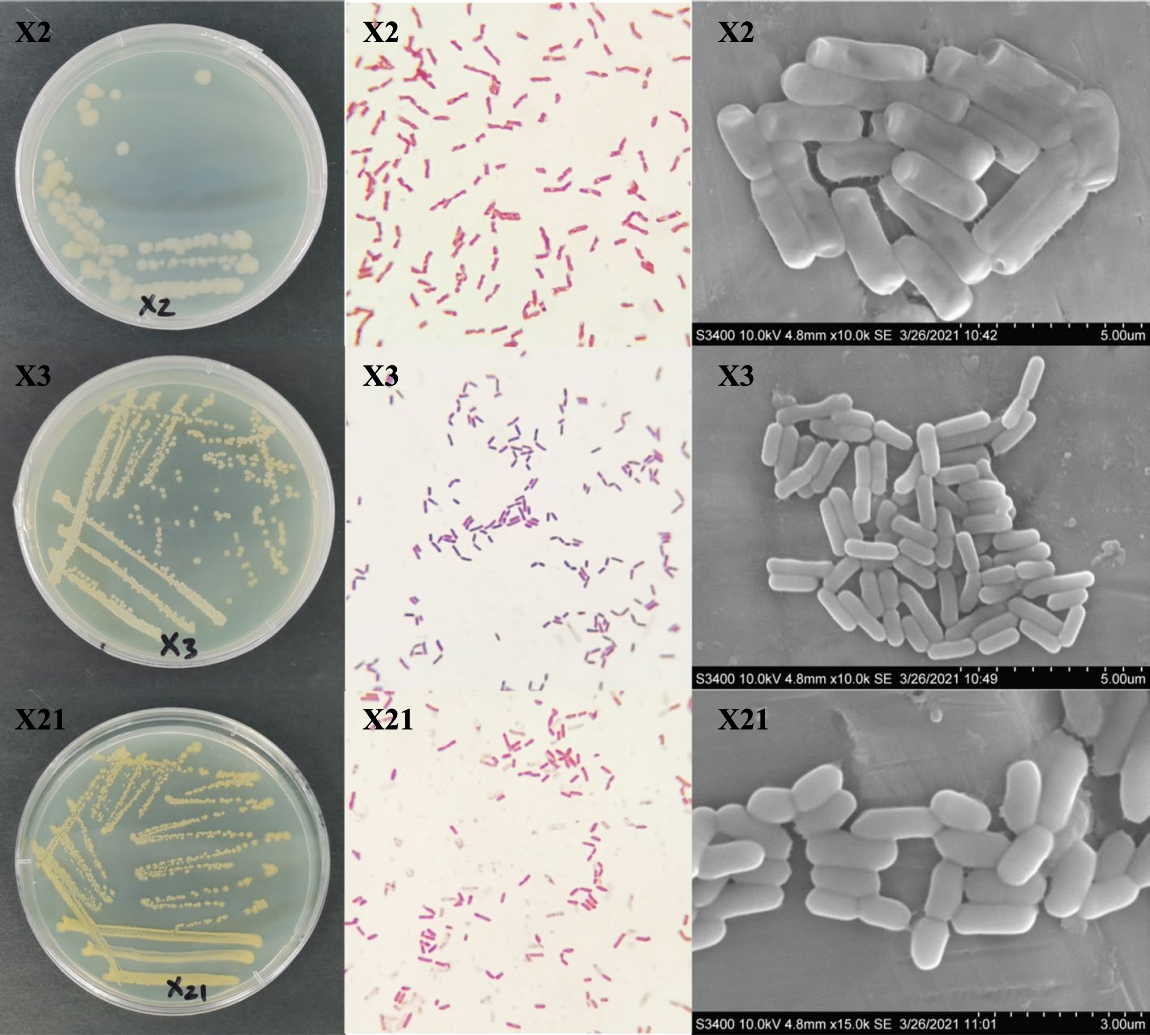
The morphology of the bacterial colonies, gram staining by SEM (× 10.^4^) of different strains (*P. moraviensis*, *B. safensis*, *F. pallidus*)

The shape and color of each strain formed similar colonies that were yellow, opaque, glossy, and orderly (Fig. 1). Scanning Electron Microscope (SEM) observation found that the thallus of X2, X3, and X21 was rhabditiform with a size of 8.3–13.5 μm × 21.7–41.9 μm, 4.1–5.2 μm × 10.1–19.9 μm, 5.1–6.9 μm × 9.0–17.1 μm, respectively. Strain X3 was Gram-positive, X2 and X21 strains were Gram-negative. X2 and X3 were facultatively anaerobic, the contact enzyme test, Voges-Proskauer (V-P) test, starch hydrolysis test, gelatin liquefaction, and nitrate reduction showed positive on X2 and X3, while the methyl red reaction (M.R) and citrate utilization test showed negative. On the contrary, strain X21 was aerobic, citrate utilization test showed positive on X21, while all the other indexes showed negative (Table 1). We also found that the sequence of X2, X3, and X21 showed 99.50%, 99.93%, and 99.86% similarity with *Pseudomonas moraviensis* CCM7280 (AY970952) (Fig. S2A), *Bacillus safensis* F-036b (AF234854) (Fig. S2B) and *Falsibacillus pallidus* CW7 (EU364818) (Fig. S2C), respectively. The obtained nucleotide sequences of X2, X3, and X21 were submitted to National Center for Biotechnology Information (NCBI) GenBank under Accession No. MZ674176, MZ674177 and MZ674178, respectively.

**Table 1:** Physiological and biochemical characteristics of strain X2, X3, X21

Project	Gram Staining	Aerobic test	Contact enzyme test	M.R test	V-Ptest	Starch hydrolysis	Gelatin Liquefaction	Nitrate reduction	Citrate Utilization
X2	-	Facultative anaerobic	+	-	+	+	+	+	-
X3	+	Aerobic or facultative anaerobic	+	-	+	+	+	+	-
X21	-	Obligate aerobic	-	-	-	-	-	-	+

Note: + :positive reaction; -:negative reaction

Based on the morphological, physiological, biochemical, and molecular characterization, X2, X3, and X21 were identified as *Pseudomonas moraviensis*, *Bacillus safensis*, and *Falsibacillus pallidus*, respectively.

### P solubilizing capacity of PSB under different culture conditions

The results showed that glucose and peptone were the best carbon and nitrogen source for assisting the P solubilizing capacity of X2 and X21, while maltose and yeast extract were the best carbon and nitrogen source for X3 (Fig. S3A, B). With the extension of the culture time, the concentration of AP in the medium that inoculated with X2, X3, and X21 rapidly reached 386, 445, and 377 mg L^−1^ on day one and then increased slowly to their maximum at day five (Fig. S3C). The greatest AP concentration of X2, X3, and X21 in the medium were all obtained at 30 ℃ and pH 6 (Fig. S3D, E). The highest AP concentration of X2, X3, and X21 in the medium with different liquid volumes per 250 mL were obtained in 100, 50, and 25 mL, respectively (Fig. S3F).

### Soil P fraction components in sandy fluvo-aquic soil of pot experiment

**Fig. 2:**
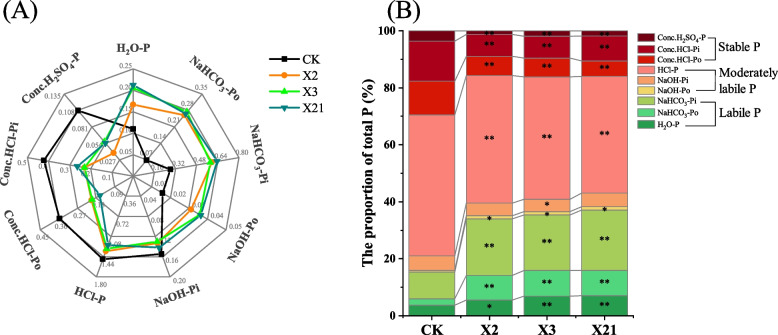
The soil phosphorus content (mg kg.^−1^) and distribution (%) of different phosphorus fraction components (H_2_O-P, NaHCO_3_-Po, NaHCO_3_-Pi, NaOH-Po, NaOH-Pi, dilute HCl-P, concentrated HCl-Po, concentrated HCl-Pi, concentrated H_2_SO_4_-P) among different treatments (CK: soil; *P. moraviensis*: soil incubated with strain *P. moraviensis*; *B. safensis*: soil incubated with strain *B. safensis*; *F. pallidus*: soil incubated with strain *F. pallidus*) in pot experiment. Compared with CK, the asterisk on the same phosphorus fraction components of each strain showed significant differences (* *P* < 0.05, ** *P* < 0.01)

Compared with the control treatment (CK), the total LP content increased by 122.04%-142.17% (*P* < 0.05) with X2, X3, and X21 inoculation, and the total MLP and SP content were significantly reduced by 8.95%–14.80% and 45.35%–46.89% (*P* < 0.05), respectively (Fig. 2A). After the inoculation with X2, X3, and X21, the content of H_2_O-P, NaHCO_3_-Po, NaHCO_3_-Pi, and NaOH-Po were increased by 51.27%–92.72%, 282.12%–302.32%, 106.90%–125.24%, and 97.47%–131.01% (*P* < 0.05), while the content of NaOH-Pi, dilute HCl-P, concentrated HCl-Po, concentrated HCl-Pi, and concentrated H_2_SO_4_-P were significantly reduced by 8.09%–15.91%, 9.57%–17.51%, 43.23%–54.74%, 37.53%–45.54%, and 47.68%–64.66% (*P* < 0.05), respectively (Fig. 2A). Moreover, we found that the proportion of the LP fractions after inoculation with PSB was significantly higher than that of CK (15.33%), which was reaching 34.05%–37.09% (*P* < 0.05). The proportion of the MLP fractions declined from 55.14% to 46.94%–50.25% and the SP fractions declined from 29.53% to 15.69%–16.12%, respectively (Fig. 2B).

### Effect of PSB on root architecture and plant growth of wheat (*Triticum aestivum*) in pot experiment

**Fig. 3:**
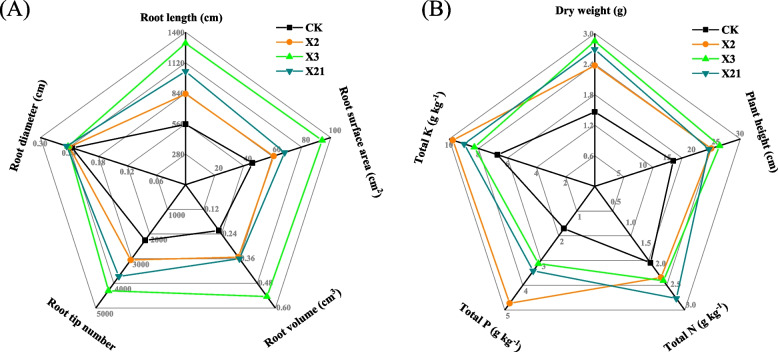
The effects of different treatments (CK: soil; *P. moraviensis*: soil incubated with strain *P. moraviensis*; *B. safensis*: soil incubated with strain *B. safensis*; *F. pallidus*: soil incubated with strain *F. pallidus*) on the root architecture (**A**) and growth (**B**) of wheat in the pot experiment

Compared with CK, the root length, root surface area, root volume, and root tip number with X2, X3, and X21 inoculation were significantly increased by 50.29%–134.09%, 31.84%–104.32%, 57.91%–143.73%, and 35.15%–90.31% (*P* < 0.05), among which inoculation with X3 performed best in enhancing the root growth. Inoculation with X21 significantly increased the root diameter by 5.06% (*P* < 0.05) (Fig. 3A). The results of root differences showed that inoculating PSB made the root grow longer and thicker, and the surface area and volumes go larger, which could be beneficial for wheat root to absorb water and nutrients in soils.

Compared with CK, the dry weight, plant height, and total NPK of inoculated PSB were significantly increased by 62.33%–95.21%, 42.62%–55.99%, 118.82%–146.24%, 83.04%–176.61% and 23.95%–45.96% (*P* < 0.05), respectively (Fig. 3B).

### Principle Component Analysis (PCA) and cluster analysis of root architecture and plant growth of wheat in pot experiment

**Fig. 4:**
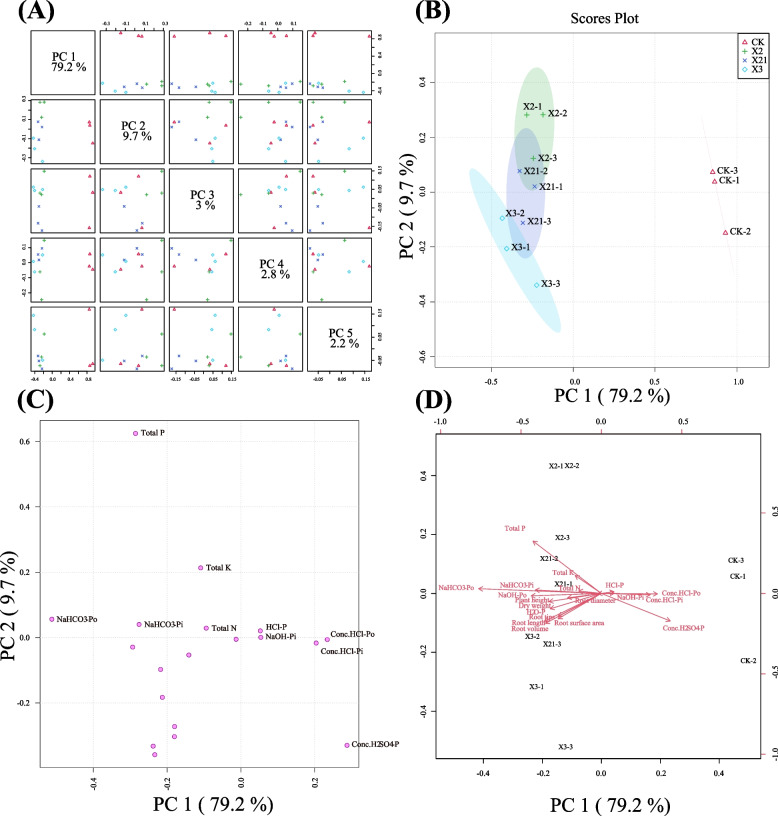
Principal component analysis (PCA) showing (**A**) pair plot, (**B**) score plot, (**C**) loading plot and (**D**) biplot (score and loading) of different attributes of the treatments (CK: soil; *P. moraviensis*: soil incubated with strain *P. moraviensis*; *B. safensis*: soil incubated with strain *B. safensis*; *F. pallidus*: soil incubated with strain *F. pallidus*) on soil phosphorus fraction components, wheat root architecture and growth in the pot experiment. Pair plot represents different explained variance ratio. Score plot represents separation of treatments. Loading plot shows the loading of each studied variable (arrows) and the arrow length approximates their variance to PC1 and PC2, whereas the angles between them represent their correlation. PC1, first principal component; PC2, second principal component

PCA results showed that PSB significantly affected the soil P fractions, root architecture, and plant growth (Fig. 4). PCA pair plot given in Fig. 4A showed that PC1, PC2, PC3, PC4, and PC5 contributing 79.2%, 9.7%, 3%, 2.8% and 2.2% respectively to the total variance. The PCA score plot explained the contribution of CK to PC1, while X2 treatment clearly contribute to PC2. X3 treatment showed no significance with both PC1 and PC2, which refers to PC2 separated the samples of CK and X21 treatments (Fig. 4B). The loading plot explained that soil concentrated HCl-Po, concentrated HCl-Pi, and concentrated H_2_SO_4_-P contributed to PC1, whereas soil NaHCO_3_-Po, NaHCO_3_-Pi, and wheat total NPK contributed to PC2. Soil NaOH-Pi and dilute HCl-P contributed to both PC1 and PC2 (Fig. 4C, D).

**Fig. 5:**
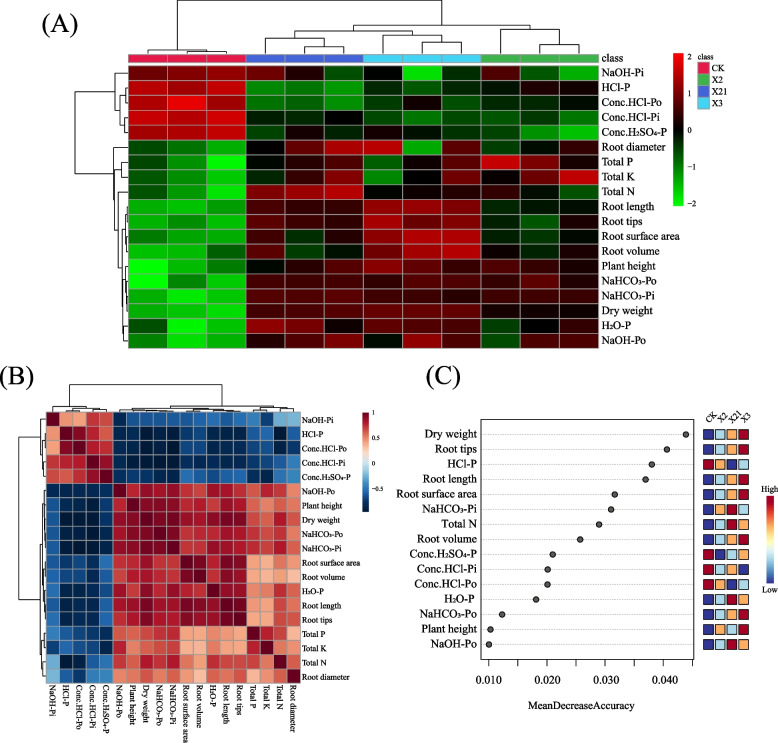
Analysis of the effect of different treatments (CK: soil; *P. moraviensis*: soil incubated with strain *P. moraviensis*; *B. safensis*: soil incubated with strain *B. safensis*; *F. pallidus*: soil incubated with strain *F. pallidus*) on soil phosphorus fraction components, wheat root architecture and growth in the pot experiment by cluster analysis. **A** heatmap analysis of soil phosphorus fraction components, wheat root architecture and growth indices; (**B**) correlation matrix showing correlation among the soil phosphorus fraction components, wheat root architecture and growth indices; (**C**) Random forest variable importance measure was used to filter the vital characteristic variables featuring different indices

Heatmap (Fig. 5A) and correlation analysis (Fig. 5B) indicated that wheat root architecture and growth-related factors showed a positive correlation with H_2_O-P, NaHCO_3_-Po, NaHCO_3_-Pi, and NaOH-Po, while a negative correlation with NaOH-Pi, dilute HCl-P, concentrated HCl-Po, concentrated HCl-Pi, and concentrated H_2_SO_4_-P. Random forest variable importance measure (Fig. 5C) indicated that for wheat growth, the values of mean in descending order were the dry weight, total N, and plant height. For root architecture, the values of mean in descending order were root tips, root length, root surface area, and root volume. For soil P fraction components, the values of mean in descending order were dilute HCl-P, NaHCO_3_-Pi, concentrated H_2_SO_4_-P, concentrated HCl-Pi, concentrated HCl-Po, H_2_O-P, NaHCO_3_-Po, and NaOH-Po.

We found H_2_O-P, NaHCO_3_-Po, NaHCO_3_-Pi, and NaOH-Po showed a positive correlation with wheat growth factors under PSB inoculation, while NaOH-Pi, dilute HCl-P, concentrated HCl-Po, concentrated HCl-Pi, and concentrated H_2_SO_4_-P showed a negative correlation with wheat growth factors (Fig. S4).

### Soil microbial biomass and activity in field under different treatments

Microbial biomass carbon (MBC) and Microbial biomass nitrogen (MBN) contents showed differently along with each growth stage of wheat (Fig. S5A, B). There were no significant differences between farmer conventional fertilization control (FP) and bone meal application control (BM) treatments in each sampling period (*P* > 0.05), indicating that bone meal has no obvious effect on soil microbial biomass. In the wheat seedling growth stage, the MBC and MBN contents with X2 and X3 inoculation were significantly higher than those of the two controls (FP and BM) (*P* < 0.05). In the wheat stem elongation stage and ripening stage, the application of three PSB strains could significantly improve the soil microbial biomass (*P* < 0.05). Furthermore, during the whole growth period of wheat, inoculation with X2, X3 and X21 significantly improved the soil base respiration (SBR) compared with controls (*P* < 0.05). The microbial metabolic quotient (qCO_2_) was significantly influenced by the inoculation with PSB at the stem elongation stage and ripening stage although not at the seedling growth stage (Fig. S5C, D).

### Soil AP and IAA content in field under different treatments

**Fig. 6:**
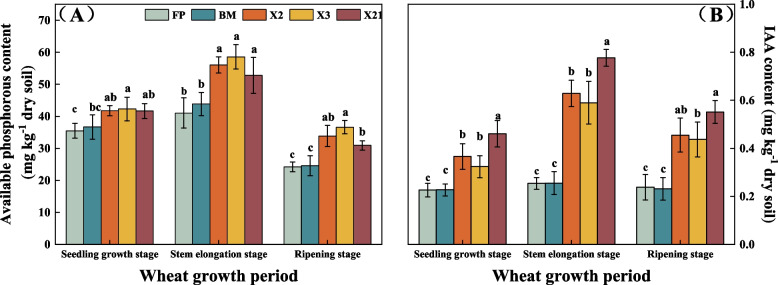
Effects of different treatments (FP: farmer conventional fertilization control; BM: bone meal control application control; *P. moraviensis*: bacteria agent *P. moraviensis*; *B. safensis*: bacteria agent *B. safensis*; *F. pallidus*: bacteria agent *F. pallidus*) on soil available phosphorous (**A**) and IAA content (**B**) at different wheat growth period in the field experiment. Different lowercase letters indicate significance among different treatments at the same stage at the 5% level

In the wheat seedling growth stage, compared with FP treatment, X3 treatment significantly increased soil AP content (*P* < 0.05). In the wheat stem elongation stage and ripening stage, the application of three PSB significantly increased AP content (*P* < 0.05) compared with FP and BM (Fig. 6A). During the whole wheat growth period, inoculation with PSB could improve the content of soil IAA content, and the application of X21 treatment showed significantly higher than all other treatments at seedling and stem elongation stage, and higher than all treatments except X2 treatment at ripening stage(Fig. 6B).

### Wheat yield and yield components in field under different treatments

Compared with FP and BM, the above-ground biomass increased by 66.97%–99.06% with PSB inoculation, the spike number increased by 44.90%–67.73% and 1000-grain weights increased by 6.92%–20.09% (*P* < 0.05). For grain number per spike, there was no significant difference among the treatments (*P* > 0.05). Compared with FP treatment, the yield increase rates of inoculation with X2, X3, and X21 were 11.18%, 14.42%, and 13.21%, respectively (Table 2).

**Table 2:** Influences of different treatments (FP: farmer conventional fertilization control; BM: bone meal application control; X2: bacteria agent X2; X3: bacteria agent X3; X21: bacteria agent X21) to wheat yield components. Data are shown as Mean ± SD. Different lowercase letters indicate significance among different treatments at the 5% level

Treatments	Aboveground biomass(kg hm^−1^)	Spike number (× 10^5^ hm^−1^)	Grain number per spike	1000-grain weights (g)	Yield(kg hm^−1^)	Yield increase rate (%)
FP	8213.98 ± 327.85b	28.30 ± 1.11c	35.74 ± 0.30a	40.22 ± 1.44c	5648.50 ± 90.15b	0
BM	8663.97 ± 327.49b	30.37 ± 1.40c	35.98 ± 1.24a	41.54 ± 0.81c	5695.64 ± 72.67b	0.83
X2	14,466.32 ± 1799.52a	44.00 ± 1.66b	36.17 ± 0.22a	44.42 ± 0.60b	6279.82 ± 103.35a	11.18
X3	16,350.46 ± 1425.65a	47.47 ± 1.96a	37.19 ± 1.55a	48.30 ± 1.84a	6463.17 ± 187.35a	14.42
X21	14,973.23 ± 1432.44a	45.57 ± 1.37ab	36.46 ± 0.49a	46.65 ± 1.24ab	6394.63 ± 159.27a	13.21

### PCA and cluster analysis of wheat yield and yield components in response to the soil microbial biomass and activity, AP and IAA content under different PSB treatments in field

PCA results showed that the three PSB significantly affected the soil microbial biomass and activity, AP and IAA content, wheat yield, and yield components (Fig. S6). PC1, PC2, PC3, PC4, and PC5 contributing 85.2%, 5.3%, 2.9%, 1.8%, and 1.2% respectively to the total variance (Fig. S6A). The PCA score plot explained the contribution of FP and BM treatments to PC1, while the X3 treatment clearly contribute to PC2. X21 treatment showed no significant contribution to both PC1 and PC2. The PC2 separated the samples of FP, BM, and X2 treatments (Fig. S6B). The loading plot explained that MBC, MBN, SBR, and AP at seedling growth period, MBN, SBR, qCO_2_, and AP at stem elongation period, MBC, MBN, AP, 1000-grain weights, and yield at the ripening period significantly contributed to PC2 (Fig. S6C).

**Fig. 7:**
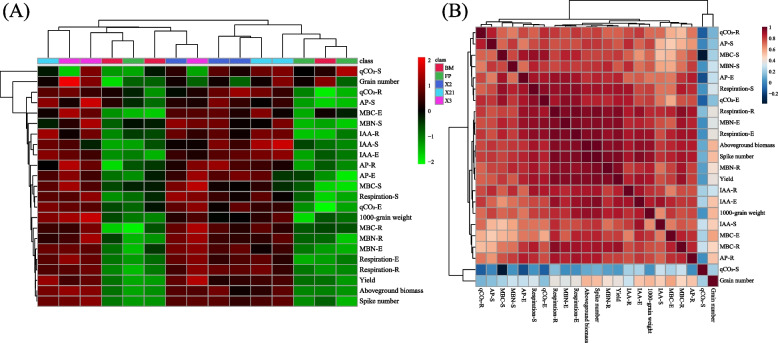
Analysis of the effect of different treatments (FP: farmer conventional fertilization control; BM: bone meal application control; *P. moraviensis*: bacteria agent *P. moraviensis*; *B. safensis*: bacteria agent *B. safensis*; *F. pallidus*: bacteria agent *F. pallidus*) on soil MBC, MBN, respiration, qCO_2_, AP, IAA content and wheat yield indices at different wheat growth period in the field experiment by cluster analysis. **A** heatmap analysis of soil MBC, MBN, respiration, qCO_2_, AP, IAA content and wheat yield indices at different wheat growth period; (**B**) correlation matrix showing correlation among the soil MBC, MBN, respiration, qCO_2_, AP, IAA content and wheat yield indices at different wheat growth period. The abbreviations are as follows: MBC: microbial biomass carbon; MBN: microbial biomass nitrogen; qCO_2_: microbial metabolic quotient; AP: soil available phosphorous; IAA: indole-3-acetic acid; S: seedling growth stage; E: stem elongation stage; R: ripening stage

Heatmap (Fig. 7A) and correlation analysis (Fig. 7B) indicated that wheat yield components (aboveground biomass, spike number, 1000-grain weights, and yield) showed a significant positive correlation with MBC, MBN, SBR, AP, and IAA in the whole wheat growth period, and qCO_2_ at stem elongation and ripening periods.

## Discussion

*P. moraviensis*, *B. safensis*, and *F. pallidus* isolated in this work were PSB that can solubilize P and produce IAA from sandy fluvo-aquic soil. Previous studies showed that some PSB can produce IAA in their metabolism to enhance plant growth and P cycling in soils [25–27]. *P. moraviensis*, *B. safensis*, and *F. pallidus* showed the highest ability of insoluble phosphate solubilization and IAA production among 40 strains isolated from soil samples. The compound of ions and PO_4_^2−^ has been found one of the main forms of fixed P in soils, which is unavailable for plants [28, 29]. At soils with pH smaller than 5.5, P precipitates with both Al and Fe ions, whereas at a pH above 7, P precipitates with Ca ions [29]. The pH value in soils from this study was 7.37 and Ca_3_(PO_4_)_2_ was selected as the phosphate resource. The concentration of phosphate solubilized by *P. moraviensis*, *B. safensis*, and *F. pallidus* reached the highest as 401.25, 476.60, and 421.15 mg L^−1^, respectively (P source 5000 mg L^−1^ Ca_3_(PO_4_)_2_), while the solubilized phosphate reached 8.02%, 9.53%, and 8.42% respectively. The large quantities of phosphate solubilized by *P. moraviensis*, *B. safensis*, and *F. pallidus* make them powerful tools to release fixed P in soil systems, which is of significant value to promote grain yield [30].

Different P fractions drive P cycling and uptake of plants in most soils, labile and some moderately labile P are easy for plants to absorb, which are key to plant root development, and further makes better growth of above-ground parts of plants [6, 31, 32]. We measured the P content in different fractions from soils after inoculation with *P. moraviensis*, *B. safensis*, and *F. pallidus* in pot experiment and found that the LP fraction increased while SP fraction declined, which is important for plant root promotion [24, 33]. In addition, the increase of LP fraction and reduction of SP fraction can be potentially due to the production of phytase/phosphatase enzymes that hydrolyze the phosphate organic compounds and secrete organic anions (such as gluconic acid) chelated with phosphate [34, 35].

The diversity and function of PSB have been found highly associated with environmental abiotic conditions, such as pH, temperature, stoichiometric allocation, and artificial interaction [36–38]. Based on cultivation and observation, we found the selected PSB reached the highest P solubilization in around 5 days of growth and their ability of P solubilization was strong under most natural soil temperatures and pH. It has been reported that *Bacillus firmus* MAJ PSB12 collected from the rhizosphere soil of the castor-oil plant showed high efficiency in solubilizing insoluble calcium triphosphate by producing a variety of organic acids, resulting in a decrease in the pH of the medium. Moreover, it could adapt to pH 5.4 and also be functional [32]. Our results confirmed that the environmental factors can interact with the biological processes of PSB and regulate their function of P solubilization. Understanding this Bio/Eco relationship would provide a foundation for selecting appropriate PSB strains that adapt to the different types of farmland under various artificial interactions [39].

The field experiment showed that *P. moraviensis*, *B. safensis*, and *F. pallidus* improved the soil microbial biomass, which potentially suggests that the PSB we investigated could survive better in wheat field and perform good cooperation with indigenous microorganisms. The soil microbial biomass has been regarded as a sensitive indicator of environmental changes [40], particularly, soil MBC that reflects the changes of soil microorganisms and their ability to act on the environment, indicating the accumulation of soil organic matter, soil MBN has a short cycle and easy mineralization [41], which is of great significance to the supply and circulation of nitrogen in the soil [42]. It has been reported that low activity and poor colonization of PSB occur after inoculating them to farmland soils, although the P solubilizing efficiency of PSB shows high in pot experiments [43]. We found that PSB identified in the present study set their population in a short time and active P solubilization productively by releasing phosphate from fixed P in soils and also promotes the P fertilizer efficiency for crops.

Besides increasing the content of LP fraction, PSB in this study can produce IAA and jointly promote the yield of crops by increasing above-ground biomass, spike number, and 1000-grain weights of wheat. Therefore, *P. moraviensis*, *B. safensis*, and *F. pallidus* showed a higher adaptation and colonization in the field under long-term fertilizer application than other bacteria isolated from rhizosphere soils [44].

## Conclusions

**Fig. 8:**
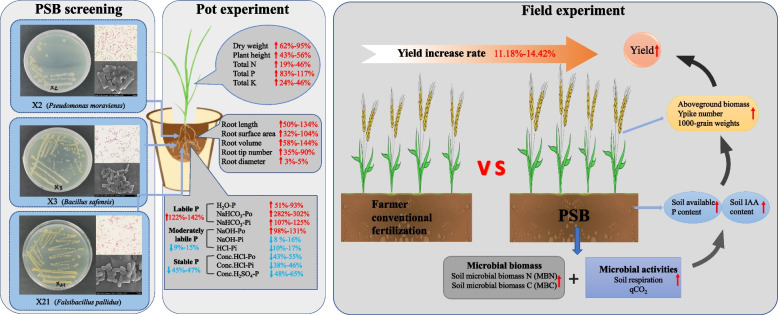
Schematic diagram of the selection of phosphate-solubilizing bacteria (PSB), the growth-promoting mechanism of wheat potted plants and the effect of wheat field application

*P. moraviensis*, *B. safensis* and *F. pallidus* isolated in our study showed superior ability to promote plant growth and high adaptation to different soil environmental conditions. We optimized the P solubilizing abiotic conditions and explored the P solubilizing process of PSB in soils. Pot experiments show that PSB can solubilize the SP and MLP fraction in the soil and PSB in the field experiments increased the utilization rate of P fertilizer. The PSB inoculation promote the wheat growth promotion and increased the crop productivity. Our work preliminary fills the gap in understanding the mechanisms of interaction between microbes and plants in the natural soil systems (Fig. 8).

## Materials and methods

### Soil sample

The sandy fluvo-aquic soil was sampled from Wheat–Maize Rotation Nutritional Fertilization Scientific Observation Station in the North China Region of the Ministry of Agriculture (34°08′15.94″North, 113°48′10.89″East) (Zhengzhou city, Henan, China). The samples were collected at 0–20 cm depth, in which the rocks, stubble, and other debris were removed. After that, soils were passed through a 2 mm sieve and divided into two parts, and placed into separate sterile bags. One group was kept for PSB screening and the other was air-dried for basic physical and chemical property measurements.

The physicochemical properties of soil samples showed 8.89 g kg^−1^ of organic matter, 30.02 mg kg^−1^ of available nitrogen, the total P was 2.95 g kg^−1^ including 31.2 mg kg^−1^ of AP, the total potassium was 19.60 g kg^−1^ including 50.39 mg kg^−1^ of available potassium, soil pH (H_2_O) was 7.37.

### Screening of PSB

Lysogeny broth (LB) and Pikovskaya (PKO) mediums were used to screen PSB and test the phosphate-solubilizing capacity [45]. LB medium supplemented with L-tryptophan was used to measure the IAA production of PSB. LB medium: Peptone 10 g, Yeast extract 5 g, NaCl 10 g, distilled water 1000 mL, pH 7.0–7.2, autoclave (30 min). PKO liquid medium: NaCl 0.3 g, Glucose 10 g, KCl 0.3 g, Ca_3_(PO4)_2_ 5 g, (NH_4_)_2_SO_4_ 0.5 g, MgSO_4_· 7H_2_O 0.3 g, MnSO_4_ 0.03 g, FeSO_4_· 7H_2_O 0.03 g, distilled water 1000 mL, pH 7.0–7.2, autoclave (30 min). The solid medium: 15–20 g agar was amended to the liquid medium.

A single colony of bacteria from the soil was isolated by the dilution method on the plate [46]. Bacterial colonies that have different appearances were transferred to LB solid medium individually by streaking on a plate until a single colony appeared. Number the isolated strains and all selected colonies were stored at 4 ℃ for later use.

The selected strains were cultured on LB liquid medium at 30 ℃ in a rotary shaker (180 rpm) for 24 h (v/v) and the activated strain was then transferred into a 250 ml flask containing 100 mL sterilized PKO liquid medium. The samples were put in an incubation shaker (180 rpm) at 30 ℃ for 96 h (3 replications per strain). The 10 mL of each culture was centrifuged for 5 min at 12,000 rpm, 4 ℃, and the supernatant was collected to measure the amount of P solubilized by each strain. The concentration of AP was determined by the molybdenum antimony colorimetric assay method [47]. Strains with high P solubilization capacity were stored at 4 ℃ and used in follow-up studies. A total of 40 bacterial strains were isolated from sandy fluvo-aquic soil, labeled as X1–X40.

### IAA-producing ability determination of PSB

IAA production of PSB was measured according to the method developed by Gordon and Weber [48]. The strains were inoculated in L-tryptophan-containing (100 mg L^−1^) LB liquid medium at 30 ℃ shaker (180 rpm) for 96 h. The 2 mL of bacterial suspension was centrifuged at 12,000 rpm for 10 min and obtained the supernatant. The 100 μg mL^−1^ IAA standard solution was serially diluted to 0, 10, 20, 30, 50, and 75 μg mL^−1^. An equal volume of Salkowski colorimetric solution (50 mL 35% HClO_4_ + 1 mL 0.5 M FeCl_3_) was added. After that, the suspensions were kept in the dark for 30 min, and the absorbance values at 530 nm wavelength were measured by spectrophotometry (UV2550 spectrophotometer, Shimazu, Japan). The content of IAA per unit volume of fermentation broth was calculated according to the standard curve.

### Morphological, physiological, biochemical, and molecular characterization of PSB

The selected strains were inoculated on LB plates at 30 ℃ for 24 h. The appearance of strain colonies including size, shape, color, gloss, consistency, and transparency was observed under a microscope (SK200, Motic) and identified by the Gram staining method [49].

The purified strains were inoculated on LB medium and cultured on a shaker at 160 rpm and 30 ℃ to the logarithmic phase. The 5 mL of sample was centrifuged at 4000 rpm for 5 min. The supernatant was discarded and the precipitate was washed three times with 0.1 M Phosphate buffer, fixed with 2.5% glutaraldehyde for 3 h, washed twice with 0.1 M phosphate buffer, 10 min each time, and then washed twice with ddH_2_O. The 30%, 50%, 70%, 80%, and 90% ethanol aqueous solutions were used for gradient dehydration (15 min each). The samples were dropped onto 5 × 5 mm tinfoil paper and air-dried naturally [50]. The morphology and size of the bacterial samples were observed by SEM (S-3400 N, Hitachi) at the Central Laboratory of Henan Agricultural University.

The physiological and biochemical characteristics of each strain were analyzed by the aerobic test, contact enzyme test, M.R, V-P test, starch hydrolyzed gelatin liquefaction, nitrate reduction and citrate utilization test [49].

16S rDNA was used as a barcode gene for classifying the different PSB strains. The total genomic DNA was extracted by the method of SDS-CTAB [51]. PCR amplification was performed on the strains selected and the primers were 27F (5′-AGAGTTTGATCCTGGCTCAG-3′) and 1492R (5′-GGTTACCTTGTTACGACTT-3′) [52]. The amplification protocol was an initial denaturation at 95 ℃ for 5 min, followed by 30 cycles of denaturation at 94 ℃ for 1 min, annealing at 57 ℃ for 40 s, and primer extension at 72 ℃ for 1.5 min. This was followed by a final extension at 72 ℃ for 10 min. The PCR products were purified with 1% agarose gel electrophoresis for sequencing and analysis (Beijing Mei Yi Mei Biological Technology Co., Ltd). These sequences were blasted on the NCBI and the 16S rDNA phylogenetic tree was reconstructed by using the neighbor-joining method with a bootstrap number of 1000 in MEGA 7.0 [53]. The sequences were deposited at GenBank and the accession numbers were obtained.

### Effect of different culture conditions on the phosphate-solubilizing capacity of strains

Different carbon sources (glucose, xylose, sucrose, fructose, mannitol, lactose, maltose), nitrogen sources (ammonium nitrate, ammonium sulfate, potassium nitrate, peptone, urea, yeast extract, alanine), culturing time (1, 2, 3, 4, 5 d), temperatures (15, 20, 25, 30, 35, 40 ℃), pH (4, 5, 6, 7, 8, 9, 10) and liquid volumes (25, 50, 75, 100, 150 mL/250 mL) were set to evaluate the effect of different PSB on P solubilizing.

### Effect of PSB strains on wheat growth in greenhouse pot experiment

The sandy fluvo-aquic soil was collected from 0–20 cm depth of field soil and filtered by a sieve (2 mm). A total of 8.4 kg of soil were collected in this study and 12 repetitive pots were conducted with 700 g of soil each. The strains prepared for the pot experiment were cultured in LB medium for 48 h at 30 ℃ and shaken at 150 rpm, then the samples were centrifuged (4000 rpm, 10 min). The pellets were washed twice with sterilized water and resuspended in sterilized water to a density of 10^7^ CFU mL^−1^, then inoculated into the soil at 10^6^ CFU g^−1^ dry soil.

The treatments were marked as X2, X3, X21 which refers to inoculation with each strain, and the CK were added with sterilized water that was equivalent to each bacteria suspension (3 replicates each). The moisture was adjusted to approximately 60% of the field capacity (24.8%, w/w) [54].

Seeds of wheat were surface-sterilized with 20% H_2_O_2_ for 20 min. They were rinsed 3–5 times with sterile distilled water and dried on a sterile plate. The seeds were germinated for 2 days at 25 ℃. For each treatment, three wheat seedlings were planted in each pot one day after the bacteria were inoculated. The day of planting was marked as the first day. The pots were then placed in a greenhouse under a day/night regimen of 10/14 h at 22/19 ℃, the photon flux density was 150 mmol m^−2^ s^−1^. The pots were watered every day to maintain the soil moisture at 24.8% (60% of the field capacity).

After 30 days of planting, the content of various P components in soils (H_2_O-P (includes resin P), 0.5 M NaHCO_3_-Po, 0.5 M NaHCO_3_-Pi, 0.1 M NaOH-Po, 0.1 M NaOH-Pi, 1 M dilute HCl-P, 12 M concentrated HCl-Po, 12 M concentrated HCl-Pi, 65% concentrated H_2_SO_4_-P) were measured according to the Hedley’s method [3], which was modified by Tiessen and Moir [4]. For this purpose, 0.5 g of soil was repeatedly extracted using different extractants with increasing chemical strength. A detailed description of the fractionation procedure used in this study is provided by Niederberger et al. [55] (Tab. S1). The classification of various P components was divided into three categories including LP, MLP and SP. LP = Ʃ H_2_O-P + NaHCO_3_-Po + NaHCO_3_-Pi, MLP = Ʃ NaOH-Po + NaOH-Pi + dilute HCl-P, SP = Ʃ concentrated HCl-Po + concentrated HCl-Pi + concentrated H_2_SO_4_-P.

The wheat plants were washed with tap water to remove the soil from the roots and then stored in 70% alcohol. The root images were taken by using a scanner (LA1600 + scanner, Canada), and the root-related parameters (root length, root surface area, root volume, and root tip number, root diameter) were evaluated with Win-rhizo software (Win-rhizo2003b, Canada). The plant height (from the root neck to the top leaf tip of the seedling) and dry weight (wash and dry the roots and aboveground parts, put them into paper bags respectively, dry them in an oven till the mass becomes constant, and measure the dry mass on an electronic balance), and plant total N, P and K were determined according to the method of Cresser and Parsons [56].

### Effect of different treatments on wheat growth in field experiment

The field experiment was conducted at Wheat–Maize Rotation Nutritional Fertilization Scientific Observation Station in the North China Region of the Ministry of Agriculture (34°08′15.94″North, 113°48′10.89″East) (Zhengzhou city, Henan, China). The field experiments test site was a semi-arid, semi-humid, warm temperate monsoon climate zone, the average annual rainfall was 676 mm. The present study was mainly conducted in July–September (annual average temperature = 14.4 ℃). The soil type was sandy fluvo-aquic and wheat and corn rotation perennial farming was applied. The selected strains were made into inoculants with the bone meal as a carrier, which contains viable bacteria of 10^11^ CFU g^−1^.

A total of 5 treatments were set in the field experiment: (1) Farmer conventional fertilization control (FP), (2) Bone meal application control (BM), (3) Bacteria agent X2 (X2), (4) Bacteria agent X3 (X3), (5) Bacteria agent X21 (X21). The experiment was set for 3 repetitions and arranged in random blocks (area of 6 × 3 m^2^). FP treatment used 600 kg hm^−2^ compound fertilizer (N: P: K = 15: 15: 15) as base fertilizer, ditching and topdressing 120 kg hm^−2^ of urea at the stem elongation stage of wheat, other treatments were treated with 40 kg hm^−2^ bone meal or a microbial agent made from the same amount of bone meal (X2, X3, X21) based on base fertilizer. The tested wheat variety was Aikang 58, the sowing rate was 150 kg hm^−2^, and other management measures during the growth period were the same as those in local high-yield wheat fields.

The 0–20 cm soil samples were collected between wheat rows at the seedling growth stage, stem elongation stage, and ripening stage, respectively, to determine soil microbes and nutrient indexes. The MBC and MBN were determined by using a chloroform fumigation-direct extraction method [57, 58]. SBR was measured by using a gas chromatography system (GC-2014, Shimadzu, Kyoto, Japan) based on the linear increase in gas with time [59]. The microbial metabolic quotient (qCO_2_ = SBR/MBC) was calculated with the formula of Anderson and Domsch [60]. Soil AP was extracted with 0.5 mol L^−1^ NaHCO_3_ and measured by the ammonium molybdate method [61]. Soil IAA concentration was determined with the high-performance liquid chromatography (HPLC) method [54]. At the mature period, the number of ears in one-meter double rows was taken, and 20 wheat plants were taken to determine the aboveground biomass, spike number, grain number per spike, 1000-grain weights, respectively. Each plot was harvested with 4 m^2^ to measure the actual yield.

### Statistical analyses

The significance of differences among treatments was analyzed by one-way ANOVA with a least significant difference (LSD) test (*P* < 0.05). Pearson correlation analyses were performed to determine the correlation among soil P fraction components, wheat root architecture and growth indices in the pot experiments, among soil microbial indices, soil AP and IAA contents, wheat yield indices in the field experiment. All of the statistical analyses were performed in SPSS 16.0 (SPSS Inc., Chicago, IL, USA). All the data were log-transformed before PCA (Metabo Analyst 5.0) analysis. ClustVis was employed to create a PCA plot and heatmaps [62]. All the graphs were generated by Origin 2018 (OriginLab Corporation, Northampton, MA, USA).

## Experimental research and field studies on plants (either cultivated or wild), including the collection of plant material

Experimental research and field studies comply with relevant institutional, national, and international guidelines and legislation.

## Supplementary Information

**Additional file 1: Tab. S1.** Sequential extraction scheme of Hedley fractionation modified by Tiessen and Moir, grouped by pools of availability.** Fig. S1.** Phosphate solubilizing and IAA producing ability of different strains (X2, X3, X21). Different lowercase letters indicate significance at the 5% level in the phosphate solubilizing ability, different uppercase letters indicate significance at the 5% level in the IAA producing ability.** Fig. S2.** (**A**) Phylogenetic trees of maximally similar species representing strains X2 (The bar represents 2 nucleotide substitutes per 1000 nucleotidesin16S rDNA sequences), (**B**) X3 (The bar represents 2 nucleotide substitutes per 1000 nucleotidesin16S rDNA sequences) and (**C**) X21 (The bar represents 5 nucleotide substitutes per 1000 nucleotidesin16S rDNA sequences).** Fig.** **S3. **Phosphate solubilizing ability of different strains (X2, X3, X21) under different carbon source (**A**), nitrogen source (**B**), incubation time (**C**), temperatures (**D**), pH values (**E**) and liquid volume (**F**). Data are shown as Mean ± SD.** Fig. S4.** Correlation analysis of each soil phosphorus fraction components correlated with the wheat root architecture and growth indices. Red means positive correlation, blue means negative correlation. * indicates significant correlation at 0.05 level, ** indicates significant correlation at 0.01 level. A-I: the compounds correlated with the H_2_O-P, NaHCO_3_-Po, NaHCO_3_-Pi, NaOH-Po, NaOH-Pi, HCl-P, Conc.HCl-Po, Conc.HCl-Pi, Conc.H_2_SO_4_-P.** Fig. S5.** Effects of different treatments (FP: farmer conventional fertilization control; BM: bone meal control application control; X2: bacteria agent X2; X3: bacteria agent X3; X21: bacteria agent X21;) on soil microbial biomass carbon (**A**), soil microbial biomass nitrogen (**B**), soil respiration (**C**) and microbial metabolic quotient (**D**) at different wheat growth period in the field experiment. Different lowercase letters indicate significance among different treatments at the same stage at the 5% level. The same below.** Fig. S6.** Principal component analysis (PCA) showing (**A**) pair plot, (**B**) score plot, (**C**) loading plot of different attributes of the treatments (FP: farmer conventional fertilization control; BM: bone meal application control; X2: bacteria agent X2; X3: bacteria agent X3; X21: bacteria agent X21) on soil MBC, MBN, respiration, qCO_2_, AP, IAA content and wheat yield indices at different wheat growth period in the field experiment. Pair plot represents different explained variance ratio. Score plot represents separation of treatments. Loading plot shows the loading indices to PC1 and PC2. PC1, first principal component; PC2, second principal component. The abbreviations are as follows: MBC: microbial biomass carbon; MBN: Microbial biomass nitrogen; qCO_2_: Microbial metabolic quotient; AP: Soil available phosphorous; IAA: Indole-3-acetic acid; S: Seedling growth stage; E: Stem elongation stage; R: Ripening stage.

## Data Availability

The datasets used and/or analysed during the current study available from the corresponding author on reasonable request.

## Abbreviations

AP: Available phosphorous
BM: Bone meal application control
CK: Control treatment
DNA: Deoxyribonucleic acid
FP: Farmer conventional fertilization control
HPLC: High-performance liquid chromatography
IAA: Indole-3-acetic acid
LB: Lysogeny broth
LP: Labile P
LSD: Least significant difference
M.R: Methyl red reaction
MBC: Microbial biomass carbon
MBN: Microbial biomass nitrogen
MLP: Moderately labile P
NCBI: National Center for Biotechnology Information
P: Phosphorus
PCA: Principal Component Analysis
PCR: Polymerase chain reaction
PKO: Pikovskaya
PSB: Phosphate-solubilizing bacteria
qCO_2_: Microbial metabolic quotient
rDNA: Ribosomal DNA
RNA: Ribonucleic acid
SBR: Soil base respiration
SEM: Scanning electron microscope
SP: Stable P
V-P: Voges-Proskauer
