# Supplementary material for: Screening of phosphate-solubilizing bacteria and their abilities of phosphorus solubilization and wheat growth promotion
Source: BMC Microbiol. 2022 Dec 9;22:296. doi: 10.1186/s12866-022-02715-7 (PMC9733106; doi:10.1186/s12866-022-02715-7)
Supplement: Supplementary file 1 — Additional file 1: Tab. S1. Sequential extraction scheme of Hedley fractionation modified by Tiessen and Moir, grouped by pools of availability. Fig. S1. Phosphate solubilizing and IAA producing ability of different strains (X2, X3, X21). Different lowercase letters indicate significance at the 5% level in the phosphate solubilizing ability, different uppercase letters indicate significance at the 5% level in the IAA producing ability. Fig. S2. (A) Phylogenetic trees of maximally similar species representing strains X2 (The bar represents 2 nucleotide substitutes per 1000 nucleotidesin16S rDNA sequences), (B) X3 (The bar represents 2 nucleotide substitutes per 1000 nucleotidesin16S rDNA sequences) and (C) X21 (The bar represents 5 nucleotide substitutes per 1000 nucleotidesin16S rDNA sequences). Fig. S3. Phosphate solubilizing ability of different strains (X2, X3, X21) under different carbon source (A), nitrogen source (B), incubation time (C), temperatures (D), pH values (E) and liquid volume (F). Data are shown as Mean ± SD. Fig. S4. Correlation analysis of each soil phosphorus fraction components correlated with the wheat root architecture and growth indices. Red means positive correlation, blue means negative correlation. * indicates significant correlation at 0.05 level, ** indicates significant correlation at 0.01 level. A-I: the compounds correlated with the H2O-P, NaHCO3-Po, NaHCO3-Pi, NaOH-Po, NaOH-Pi, HCl-P, Conc.HCl-Po, Conc.HCl-Pi, Conc.H2SO4-P. Fig. S5. Effects of different treatments (FP: farmer conventional fertilization control; BM: bone meal control application control; X2: bacteria agent X2; X3: bacteria agent X3; X21: bacteria agent X21;) on soil microbial biomass carbon (A), soil microbial biomass nitrogen (B), soil respiration (C) and microbial metabolic quotient (D) at different wheat growth period in the field experiment. Different lowercase letters indicate significance among different treatments at the same stage at the 5% level. The same be [file 12866_2022_2715_MOESM1_ESM.docx]

**Supplementary information**

**Tab. S1.** Sequential extraction scheme of Hedley fractionation modified by Tiessen and Moir, grouped by pools of availability.

| Different extractants | Fractionation procedure | Determine procedure | Modeled Fractions | Modeled Pools |
| --- | --- | --- | --- | --- |
| Resin strip in water | H_2_O + Resin | Determine Pi | H_2_O-P | Labile P fractions |
| 0.5 M NaHCO_3_ | Bicarb-extractable Pt | Digest, determine Pt |  |  |
|  | Precipitate organic matter | Determine Pi | NaHCO_3_-Pi |  |
|  |  | Po=Pt-Pi | NaHCO_3_-Po |  |
| 0.1 M NaOH | NaOH-extractable Pt | Digest, determine Pt |  | Moderately labile P fractions |
|  | Precipitate organic matter | Determine Pi | NaOH-Pi |  |
|  |  | Po=Pt-Pi | NaOH-Po |  |
| 1 M dilute HCl-P |  | Determine Pi | dilute HCl-P |  |
| 12 M concentrated HCl | HCl-extractable Pt | Digest, determine Pt |  | Stable P fractions |
|  |  | Determine Pi | concentrated HCl-Pi |  |
|  |  | Po=Pt-Pi | concentrated HCl-Po |  |
| 65% concentrated H_2_SO_4_ |  | Digest, determine Pt | concentrated H_2_SO_4_-P |  |





**Fig. S1.** Phosphate solubilizing and IAA producing ability of different strains (X2, X3, X21). Different lowercase letters indicate significance at the 5% level in the phosphate solubilizing ability, different uppercase letters indicate significance at the 5% level in the IAA producing ability.


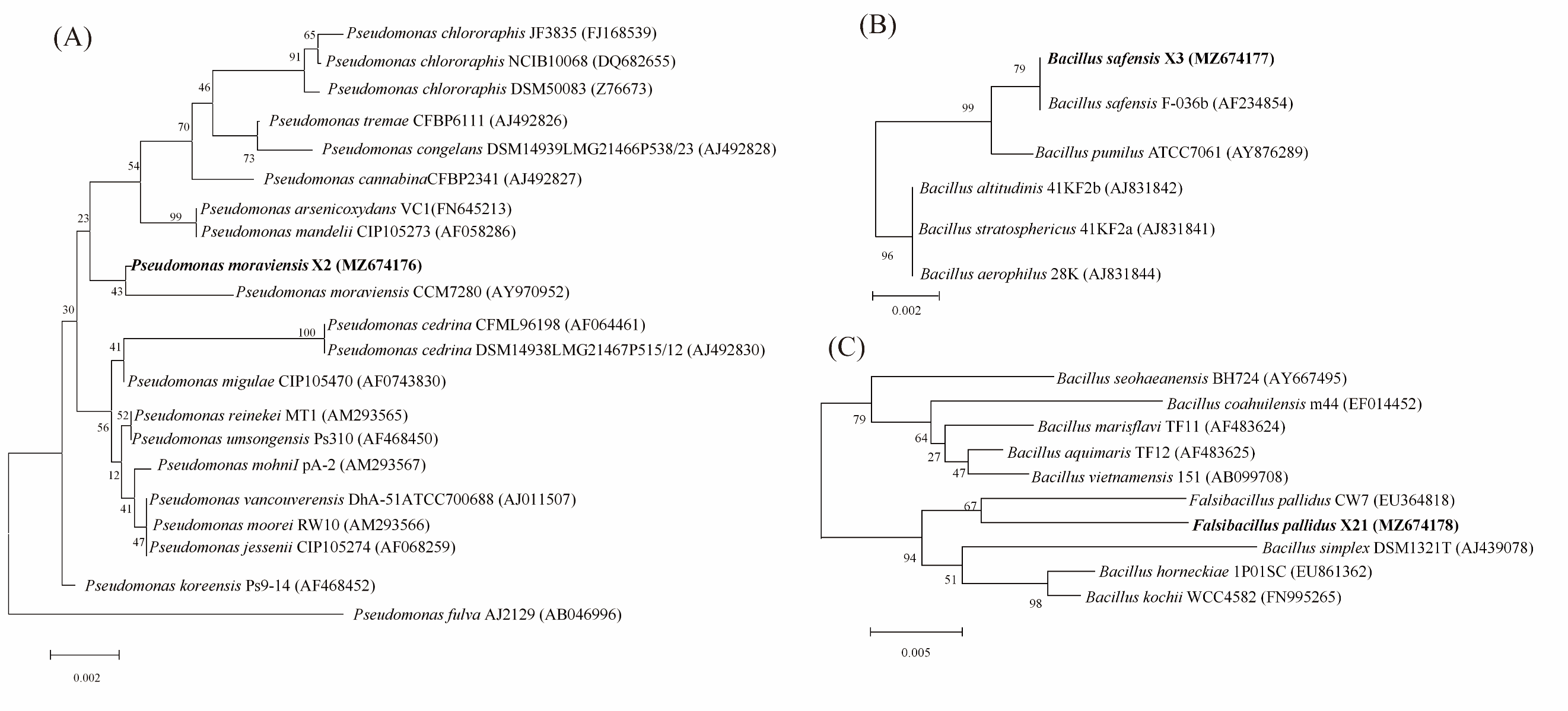


**Fig. S2.** (A) Phylogenetic trees of maximally similar species representing strains X2 (The bar represents 2 nucleotide substitutes per 1000 nucleotidesin16S rDNA sequences), (B) X3 (The bar represents 2 nucleotide substitutes per 1000 nucleotidesin16S rDNA sequences) and (C) X21 (The bar represents 5 nucleotide substitutes per 1000 nucleotidesin16S rDNA sequences).





**Fig. S3.** Phosphate solubilizing ability of different strains (X2, X3, X21) under different carbon source (A), nitrogen source (B), incubation time (C), temperatures (D), pH values (E) and liquid volume (F). Data are shown as Mean ± SD.


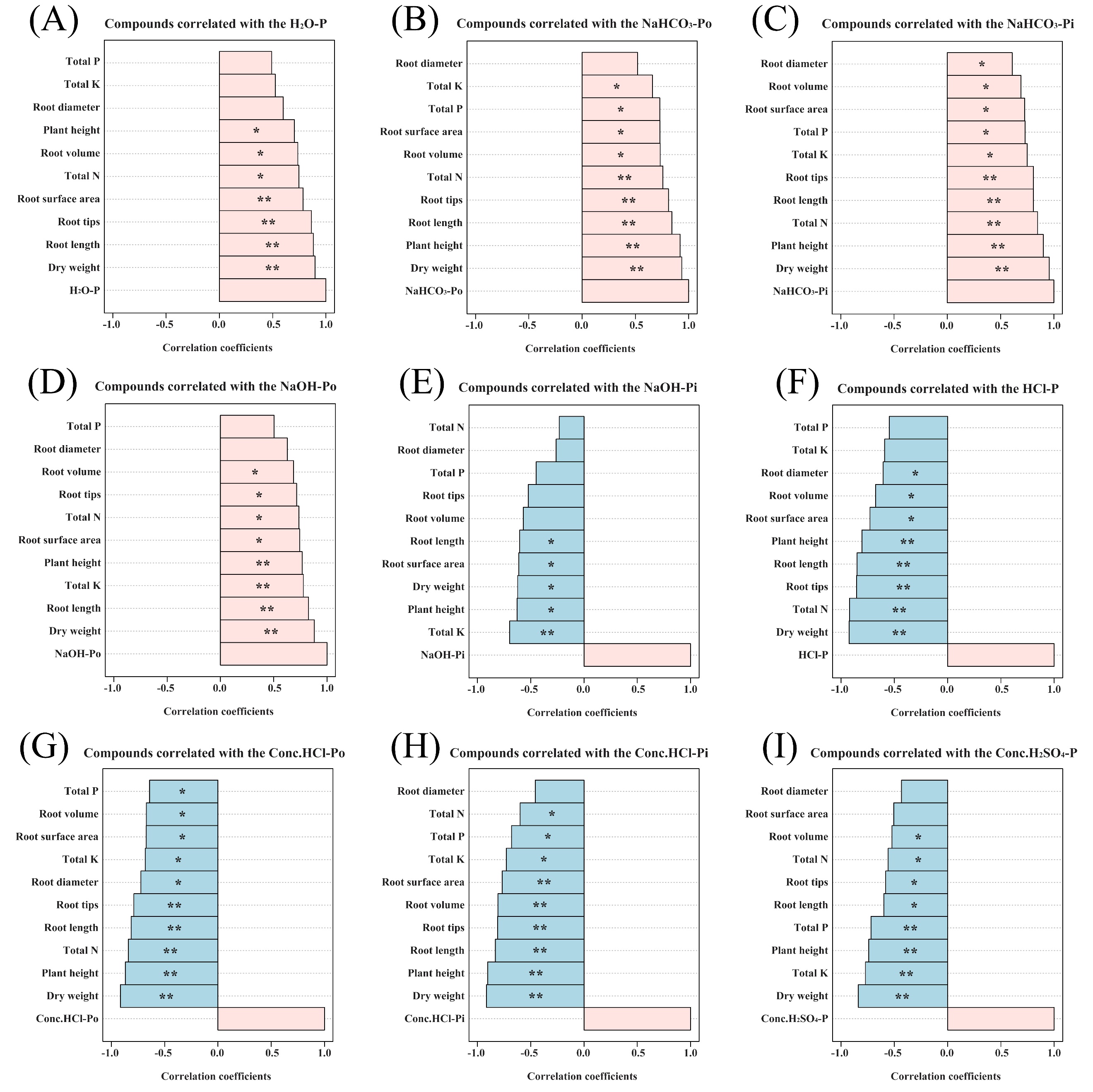


**Fig. S4.** Correlation analysis of each soil phosphorus fraction components correlated with the wheat root architecture and growth indices. Red means positive correlation, blue means negative correlation. * indicates significant correlation at 0.05 level, ** indicates significant correlation at 0.01 level. A-I: the compounds correlated with the H_2_O-P, NaHCO_3_-Po, NaHCO_3_-Pi, NaOH-Po, NaOH-Pi, HCl-P, Conc.HCl-Po, Conc.HCl-Pi, Conc.H_2_SO_4_-P.


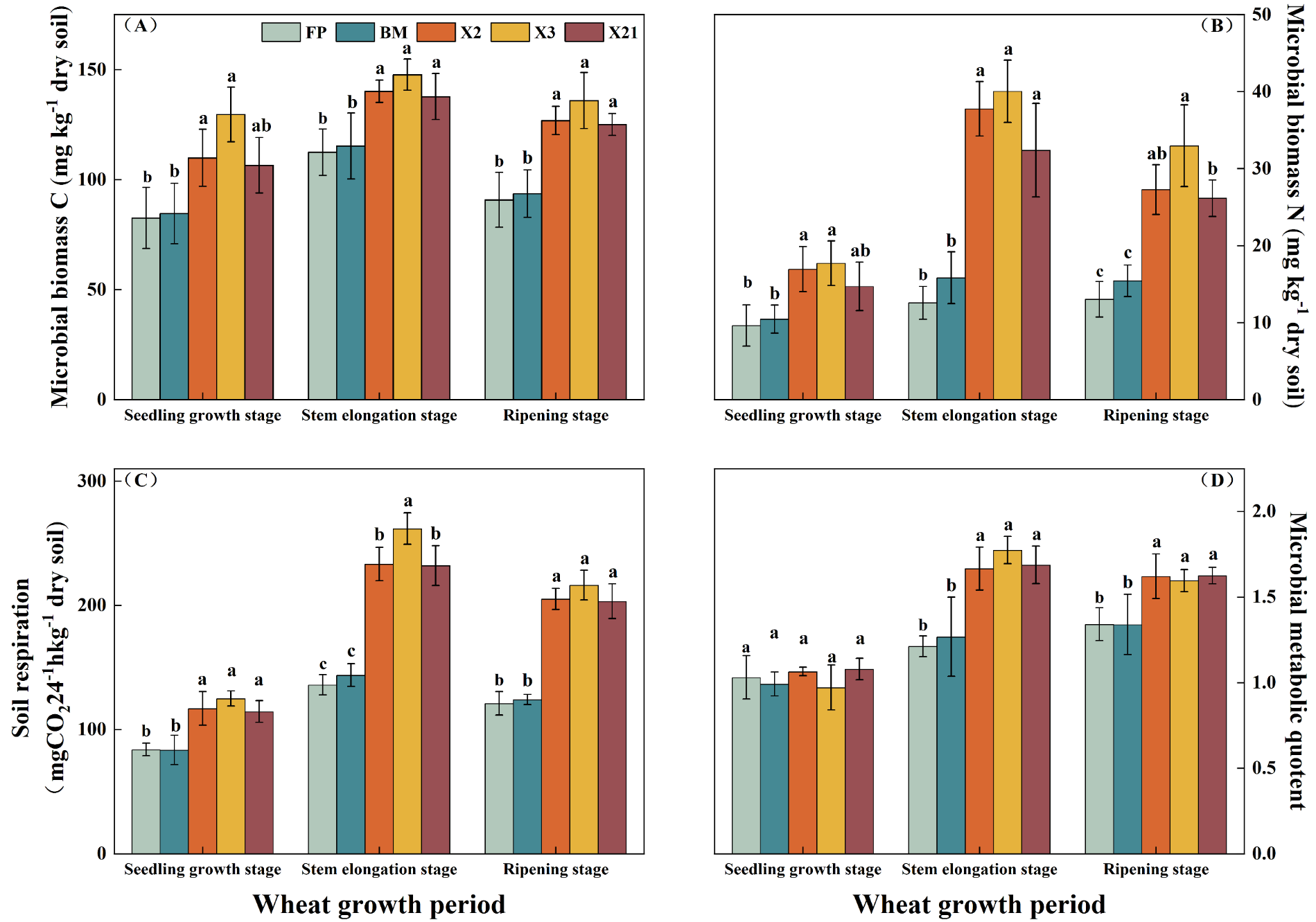


**Fig. S5.** Effects of different treatments (FP: farmer conventional fertilization control; BM: bone meal control application control; X2: bacteria agent X2; X3: bacteria agent X3; X21: bacteria agent X21;) on soil microbial biomass carbon (A), soil microbial biomass nitrogen (B), soil respiration (C) and microbial metabolic quotient (D) at different wheat growth period in the field experiment. Different lowercase letters indicate significance among different treatments at the same stage at the 5% level. The same below.


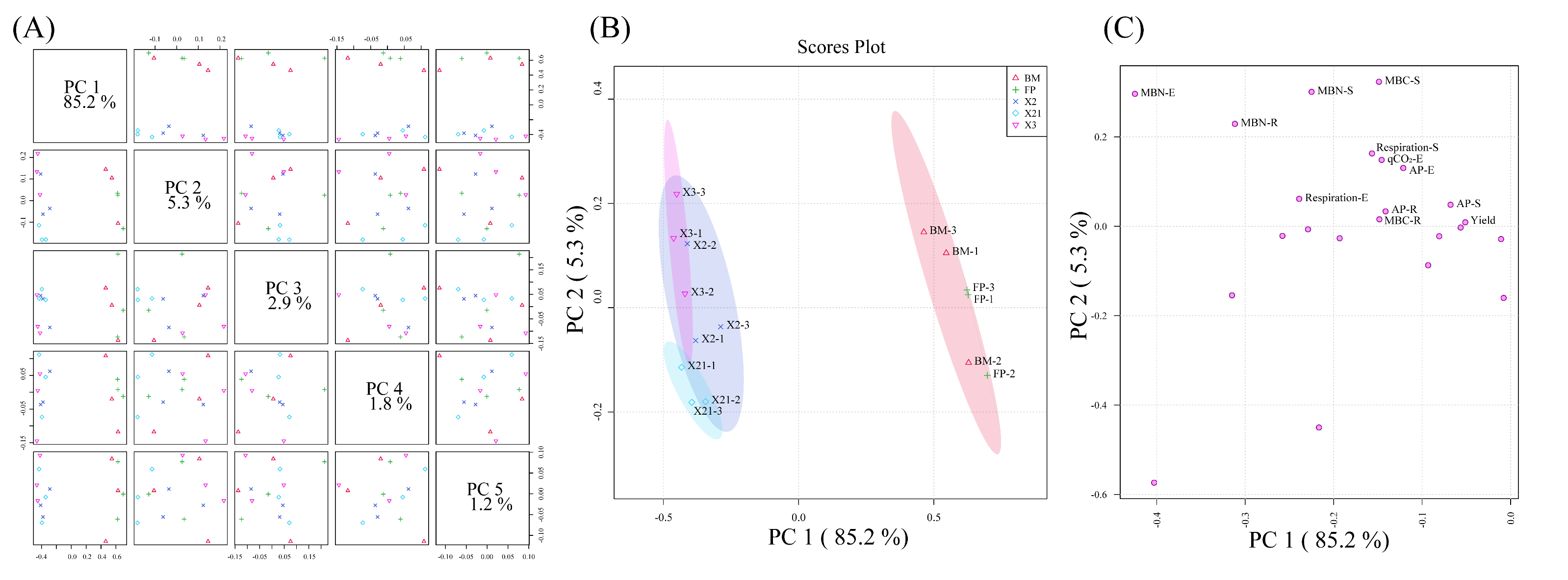


**Fig. S6.** Principal component analysis (PCA) showing (A) pair plot, (B) score plot, (C) loading plot of different attributes of the treatments (FP: farmer conventional fertilization control; BM: bone meal application control; X2: bacteria agent X2; X3: bacteria agent X3; X21: bacteria agent X21) on soil MBC, MBN, respiration, qCO_2_, AP, IAA content and wheat yield indices at different wheat growth period in the field experiment. Pair plot represents different explained variance ratio. Score plot represents separation of treatments. Loading plot shows the loading indices to PC1 and PC2. PC1, first principal component; PC2, second principal component. The abbreviations are as follows: MBC: microbial biomass carbon; MBN: Microbial biomass nitrogen; qCO_2_: Microbial metabolic quotient; AP: Soil available phosphorous; IAA: Indole-3-acetic acid; S: Seedling growth stage; E: Stem elongation stage; R: Ripening stage.
